# Independent associations of psychiatric disorders with atrial fibrillation and acute coronary syndrome: An analysis of the National Inpatient Sample

**DOI:** 10.1016/j.ijcrp.2026.200704

**Published:** 2026-08-24

**Authors:** Abdelrazig Suliman, Fayz Quadri, Gloria Elizondo, Harshil Patel, Abdullah Rabah, Chi Ayrsh Ford

**Affiliations:** Department of Internal Medicine, University of Texas Health Science Center at Tyler, Tyler, TX, USA

**Keywords:** Psychiatric disorders, Depression, Anxiety disorders, Bipolar disorder, Atrial fibrillation, Acute coronary syndrome, National inpatient sample

## Abstract

**Background:**

Psychiatric disorders are associated with adverse cardiovascular outcomes, but their independent associations with atrial fibrillation (AF) and acute coronary syndrome (ACS), accounting for psychiatric comorbidity and age-related differences, remain unclear. This study evaluated the associations of depression, anxiety disorders, and bipolar disorder with AF and ACS in hospitalized adults.

**Methods:**

We conducted a retrospective cross-sectional analysis of the 2017–2020 Healthcare Cost and Utilization Project National Inpatient Sample. Hospitalizations among adults aged 18–90 years were included. Survey-weighted multivariable logistic regression models were constructed separately for AF and ACS and adjusted for sex, race or ethnicity, calendar year, cardiovascular risk factors, and coexisting psychiatric disorders. Hypertension included essential, secondary, resistant, and crisis hypertension, as well as hypertensive heart or kidney disease. Domain analyses were performed among adults aged 18–39 years, and interactions of age group with depression, anxiety disorders, bipolar disorder, and hypertension were evaluated.

**Results:**

The age-restricted dataset contained 23,713,289 discharge records; fully adjusted complete-case models included 23,068,920 records, representing 115,344,584 weighted hospitalizations. In the full models, depression was associated with slightly higher odds of AF (adjusted OR, 1.041; 95% CI, 1.037–1.045), whereas anxiety disorders (0.970; 0.966–0.974) and bipolar disorder (0.848; 0.841–0.855) were associated with lower odds. Depression (0.711; 0.704–0.718), anxiety disorders (0.862; 0.854–0.870), and bipolar disorder (0.504; 0.492–0.516) were associated with lower odds of ACS. Hypertension was associated with higher odds of both AF (2.615; 2.598–2.633) and ACS (2.103; 2.080–2.126). Among adults aged 18–39 years, depression and anxiety disorders were associated with higher odds of AF, while bipolar disorder was not significant. All three psychiatric exposures were associated with lower odds of ACS in this subgroup. Age interactions were significant for the psychiatric exposures and hypertension for AF; for ACS, the age interactions were significant for depression, bipolar disorder, and hypertension, but not anxiety.

**Conclusions:**

Psychiatric diagnoses showed small but statistically significant, outcome- and age-dependent associations with AF and ACS after multivariable adjustment. Because these are hospitalization-level cross-sectional associations, they should not be interpreted as causal or protective. Longitudinal studies are needed to clarify the temporal and biological mechanisms underlying these findings.

## Graphical abstract

**Figure:**
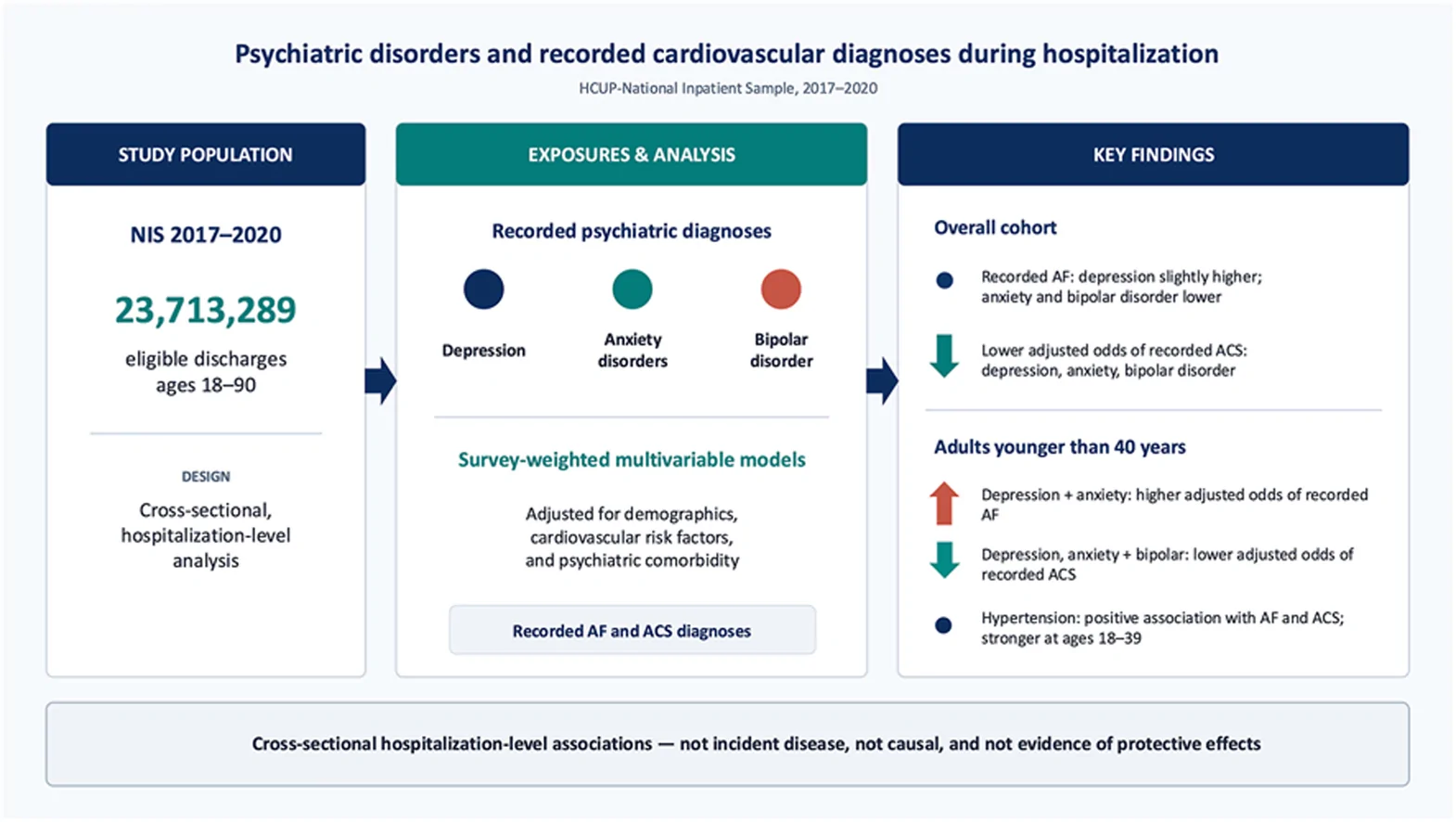

## 1 Introduction

Psychiatric disorders are leading causes of disability globally and represent a significant and growing public health concern. The Global Burden of Disease Study 2019 reported that mental disorders account for a considerable proportion of years lived with disability and continue to contribute to premature mortality and increased healthcare utilization worldwide [1]. In the United States, depression, anxiety disorders, and bipolar disorder are among the most prevalent psychiatric conditions, affecting millions of adults annually and frequently co-occurring with chronic medical illnesses [2]. Beyond their impact on emotional well-being and quality of life, psychiatric disorders are increasingly recognized as systemic conditions that influence multiple physiological pathways and contribute to adverse cardiovascular outcomes [3,4].

The association between psychiatric illness and cardiovascular disease is well established. Numerous epidemiologic studies have demonstrated that individuals with depression have higher rates of coronary artery disease, myocardial infarction, heart failure, stroke, and cardiovascular mortality than those without depression [3,5]. Similar associations have been observed for anxiety disorders, which are linked to increased risks of coronary heart disease, sudden cardiac death, and adverse cardiovascular events [6,7]. Likewise, individuals with bipolar disorder experience significantly higher rates of premature cardiovascular disease and reduced life expectancy, primarily due to cardiovascular causes [8]. Although unhealthy lifestyle behaviors, including smoking, obesity, physical inactivity, and medication nonadherence, partially explain these findings, accumulating evidence indicates that biological mechanisms also contribute substantially.

Multiple interrelated biological pathways have been proposed to explain the relationship between psychiatric disorders and cardiovascular disease. Chronic psychological stress can activate the hypothalamic–pituitary–adrenal axis and sympathetic nervous system, leading to sustained elevations in cortisol and catecholamines that promote hypertension, insulin resistance, endothelial dysfunction, and adverse cardiac remodeling [3,9]. Depression and anxiety are also associated with increased circulating inflammatory cytokines, oxidative stress, platelet activation, and vascular inflammation, all of which contribute to the development and progression of atherosclerosis and cardiac arrhythmias [[10], [11], [12]]. These biological abnormalities frequently coexist with traditional cardiovascular risk factors, further amplifying the cardiovascular risk among individuals with psychiatric disorders.

Among cardiovascular diseases, atrial fibrillation (AF) and acute coronary syndrome (ACS) are two of the most common causes of hospitalization worldwide. AF is the most frequently encountered sustained cardiac arrhythmia and is associated with increased risks of stroke, heart failure, cognitive decline, hospitalization, and mortality [13,14]. Similarly, ACS remains a leading cause of cardiovascular morbidity and mortality despite significant advances in preventive therapies and coronary revascularization [14,15]. Because both conditions share inflammatory, autonomic, and metabolic pathways with psychiatric disorders, there is growing interest in understanding their interrelationship.

Previous research has identified associations between depression, anxiety disorders, and the subsequent development of AF and coronary artery disease. Large prospective cohort studies and systematic reviews have demonstrated that depression and anxiety are associated with increased incidence of AF and ischemic heart disease, even after adjustment for conventional cardiovascular risk factors [6,13]. Additionally, depression has consistently been identified as an independent predictor of recurrent myocardial infarction, cardiovascular mortality, and adverse outcomes following ACS [3,5]. Nevertheless, these associations have not been entirely consistent across studies. Variations in study populations, outcome definitions, adjustment strategies, and follow-up durations have resulted in heterogeneous findings, indicating that the relationship between psychiatric disorders and cardiovascular disease is more complex than previously understood.

Despite the expanding literature, several important knowledge gaps remain. First, most prior investigations have assessed depression, anxiety disorders, or bipolar disorder independently, although these conditions frequently overlap in clinical practice. Given the high prevalence of psychiatric comorbidity, failure to account for coexisting psychiatric disorders may bias estimates of the independent associations between specific psychiatric conditions and cardiovascular disease. Second, most published studies have focused on incident cardiovascular events in prospective community-based cohorts, whereas comparatively less is known about the relationship between psychiatric disorders and cardiovascular diagnoses among hospitalized patients. Analyses based on hospitalized populations represent a distinct clinical context in which differences in patient characteristics, healthcare utilization, disease severity, and diagnostic coding may influence observed associations. Finally, few studies have examined whether these relationships vary by age, particularly among younger adults, despite increasing recognition that early-onset cardiovascular disease may exhibit unique clinical features and risk profiles.

To our knowledge, this study is among the first nationally representative investigations to (1) simultaneously evaluate depression, anxiety disorders, and bipolar disorder within the same multivariable models, (2) examine their independent associations with both AF and ACS, and (3) investigate age-related heterogeneity through prespecified subgroup and interaction analyses. By addressing these gaps, this study provides a more comprehensive assessment of the relationship between psychiatric disorders and major cardiovascular outcomes among hospitalized adults.

To address these gaps, we conducted a retrospective cross-sectional study using the HCUP National Inpatient Sample (NIS). We evaluated the independent associations of depression, anxiety disorders, and bipolar disorder with AF and ACS among hospitalized adults aged 18–90 years. Analyses were adjusted for demographic characteristics, calendar year, established cardiovascular risk factors, and psychiatric comorbidity. We also examined these associations among adults aged 18–39 years and assessed whether age group modified the associations of each psychiatric exposure and hypertension with the cardiovascular outcomes.

## 2 Methods

### 2.1 Study design and data source

A retrospective cross-sectional study was conducted using data from the HCUP NIS for 2017–2020. Developed by the Agency for Healthcare Research and Quality (AHRQ), the NIS contains discharge-level information from approximately 20% of community hospitalizations and provides nationally representative estimates of inpatient care through a stratified probability sampling design. Each hospitalization record includes patient demographic characteristics, hospital information, primary and secondary diagnoses, procedures, discharge disposition, and hospital charges coded using the International Classification of Diseases, Tenth Revision, Clinical Modification (ICD-10-CM) [16].

As the NIS is a hospitalization-level database, each record represents an individual hospital admission rather than a unique patient. Therefore, repeat hospitalizations by the same patient cannot be identified or linked longitudinally. This study used publicly available de-identified data and was exempt from institutional review board review and informed consent requirements. The study was conducted and reported in accordance with the Strengthening the Reporting of Observational Studies in Epidemiology (STROBE) guidelines [17].

### 2.2 Study population

Hospitalizations of adults aged 18–90 years during 2017–2020 were included. AF was identified using ICD-10-CM diagnosis code I48. ACS was defined by unstable angina (I20.0), acute myocardial infarction (I21), or subsequent myocardial infarction (I22). Each outcome was analyzed independently as a binary variable based on whether the corresponding diagnosis was recorded during the hospitalization.

### 2.3 Exposure variables and covariates

The primary exposure variables were depression, anxiety disorders, and bipolar disorder, identified using ICD-10-CM diagnosis codes. Given the frequent co-occurrence of these psychiatric disorders, they were not treated as mutually exclusive. All three psychiatric conditions were simultaneously included in the multivariable regression models to estimate the independent association of each disorder while accounting for psychiatric comorbidity.

We chose covariates ahead of time based on their clinical importance and known links to psychiatric disorders and heart disease. We mainly treated demographic factors as possible confounders. Traditional cardiovascular risk factors, such as hypertension, diabetes, obesity, smoking or tobacco use, and dyslipidemia, could act as confounders or mediators. By including these factors, we could estimate how psychiatric disorders relate to AF and ACS while taking these heart risk factors into account. Consequently, the adjusted estimates do not show the full impact of psychiatric disorders on heart health.

Demographic variables included age, sex, and race or ethnicity. Age was summarized continuously for descriptive analyses and dichotomized as 18–39 versus 40–90 years for the adjusted, subgroup, and interaction analyses.

Race and ethnicity were categorized as White, Black, Hispanic, Asian or Pacific Islander, Native American, and Other, with White patients serving as the reference group in all multivariable analyses. Traditional cardiovascular risk factors included hypertension, diabetes mellitus, obesity, smoking or tobacco use, and dyslipidemia. Hypertension was defined by any diagnosis of essential, secondary, resistant, or crisis hypertension or hypertensive heart or kidney disease (ICD-10-CM I10–I13, I15–I16, or I1A.0). These variables were treated as binary indicators of presence or absence. The ICD-10-CM codes used to define psychiatric disorders, cardiovascular outcomes, and cardiovascular risk factors are provided in Supplementary Table 1.

### 2.4 Statistical analysis

Baseline demographic and clinical characteristics were summarized by AF and ACS status. Continuous variables are reported as means with standard deviations (SDs), and categorical variables as frequencies and percentages.

The NIS discharge weight was applied to regression analyses, and all models accounted for the complex survey design using year-specific strata and hospital clusters. Baseline tables report unweighted discharge counts and column percentages. The unit of analysis was the hospitalization rather than the individual patient.

Separate multivariable logistic regression models were developed for AF and ACS to estimate the independent associations of depression, anxiety disorders, and bipolar disorder with each cardiovascular outcome. Variables included in the multivariable models were selected a priori based on established clinical relevance rather than automated selection procedures. The fully adjusted models included age group, sex, race or ethnicity, hypertension, diabetes mellitus, obesity, smoking, dyslipidemia, depression, anxiety disorders, and bipolar disorder. Adjusted odds ratios (ORs) with 95% confidence intervals (CIs) were reported.

A prespecified survey-domain analysis was conducted among adults aged 18–39 years. The subgroup models included the same covariates as the full models except age group, which was constant within the domain.

Interaction models added product terms between age group (18–39 versus 40–90 years) and depression, anxiety disorders, bipolar disorder, and hypertension to assess age-related effect modification for AF and ACS.

As the NIS is a cross-sectional administrative database without longitudinal follow-up, the analyses estimate associations between psychiatric disorders and cardiovascular diagnoses recorded during the same hospitalization. These analyses cannot establish temporal relationships or causal inferences.

All tests were two-sided, and p < 0.05 was considered statistically significant. Analyses were performed with the Complex Samples procedures in IBM SPSS Statistics using the NIS discharge weights, year-specific strata, and year-specific hospital clusters.

## 3 Results

### 3.1 Study population

The age-restricted dataset contained 23,713,289 discharge records. AF was recorded in 3,634,248 (15.3%) and ACS in 582,770 (2.5%). Because race was missing for 642,373 records and sex for 2134 records, the fully adjusted complete-case models included 23,068,920 records, representing 115,344,584 weighted hospitalizations.

### 3.2 Baseline characteristics

Hospitalizations with AF were substantially older than those without AF (mean age, 74.96 ± 11.72 versus 55.08 ± 19.92 years); 99.1% of AF hospitalizations occurred among adults aged 40–90 years. Hypertension was present in 84.6% of AF hospitalizations and 51.5% of those without AF. AF hospitalizations were also more often male and had higher prevalences of depression, anxiety disorders, dyslipidemia, diabetes mellitus, and obesity, but lower prevalences of bipolar disorder and smoking (Table 1).

**Table 1:** Baseline demographic and clinical characteristics by atrial fibrillation status.

Characteristic	No AF (N = 20,079,041)	AF (N = 3,634,248)
Age, mean (SD)	55.08 (19.92)	74.96 (11.72)
Age, median [min, max]	57 [18, 90]	77 [18, 90]
**Age group**		
18–39	5,640,796 (28.1%)	31,456 (0.9%)
40–90	14,438,245 (71.9%)	3,602,792 (99.1%)
**Race/ethnicity**		
White	12,558,115 (64.3%)	2,853,075 (80.4%)
Black	3,210,721 (16.4%)	331,895 (9.4%)
Hispanic	2,417,602 (12.4%)	206,487 (5.8%)
Asian or Pacific Islander	573,082 (2.9%)	71,143 (2.0%)
Native American	140,704 (0.7%)	13,350 (0.4%)
Other	622,892 (3.2%)	71,850 (2.0%)
Missing race	555,925 (2.8%)	86,448 (2.4%)
**Sex**		
Male	8,238,903 (41.0%)	1,933,530 (53.2%)
Female	11,838,104 (59.0%)	1,700,618 (46.8%)
Missing sex	2034 (<0.1%)	100 (<0.1%)
**Any hypertension diagnosis**		
No	9,746,482 (48.5%)	558,631 (15.4%)
Yes	10,332,559 (51.5%)	3,075,617 (84.6%)
**Diabetes mellitus**		
No	17,941,410 (89.4%)	3,148,479 (86.6%)
Yes	2,137,631 (10.6%)	485,769 (13.4%)
**Obesity**		
No	16,832,961 (83.8%)	2,946,970 (81.1%)
Yes	3,246,080 (16.2%)	687,278 (18.9%)
**Smoking/tobacco use**		
No	17,312,574 (86.2%)	3,373,528 (92.8%)
Yes	2,766,467 (13.8%)	260,720 (7.2%)
**Dyslipidemia**		
No	14,968,760 (74.5%)	1,968,101 (54.2%)
Yes	5,110,281 (25.5%)	1,666,147 (45.8%)
**Depression**		
No	17,815,322 (88.7%)	3,130,513 (86.1%)
Yes	2,263,719 (11.3%)	503,735 (13.9%)
**Anxiety disorders**		
No	17,814,666 (88.7%)	3,170,715 (87.2%)
Yes	2,264,375 (11.3%)	463,533 (12.8%)
**Bipolar disorder**		
No	19,592,974 (97.6%)	3,557,883 (97.9%)
Yes	486,067 (2.4%)	76,365 (2.1%)

Values are unweighted discharge counts (column percentages) from all eligible discharges. Race and sex missingness are shown explicitly. Hypertension includes essential, secondary, resistant, and crisis hypertension and hypertensive heart or kidney disease. AF = atrial fibrillation; ACS = acute coronary syndrome; SD = standard deviation.

Hospitalizations with ACS were older than those without ACS (mean age, 69.09 ± 13.51 versus 57.85 ± 20.27 years); 97.8% of ACS hospitalizations occurred among adults aged 40–90 years. Hypertension was present in 84.4% of ACS hospitalizations and 55.8% of those without ACS. ACS hospitalizations were also more often male and had higher prevalences of dyslipidemia, diabetes mellitus, obesity, and smoking, whereas depression, anxiety disorders, and bipolar disorder were less prevalent (Table 2).

**Table 2:** Baseline demographic and clinical characteristics by acute coronary syndrome status.

Characteristic	No ACS (N = 23,130,519)	ACS (N = 582,770)
Age, mean (SD)	57.85 (20.27)	69.09 (13.51)
Age, median [min, max]	61 [18, 90]	70 [18, 90]
**Age group**		
18–39	5,659,682 (24.5%)	12,570 (2.2%)
40–90	17,470,837 (75.5%)	570,200 (97.8%)
**Race/ethnicity**		
White	15,003,756 (66.7%)	407,434 (71.9%)
Black	3,470,195 (15.4%)	72,421 (12.8%)
Hispanic	2,573,106 (11.4%)	50,983 (9.0%)
Asian or Pacific Islander	627,770 (2.8%)	16,455 (2.9%)
Native American	150,639 (0.7%)	3415 (0.6%)
Other	678,681 (3.0%)	16,061 (2.8%)
Missing race	626,372 (2.7%)	16,001 (2.7%)
**Sex**		
Male	9,832,855 (42.5%)	339,578 (58.3%)
Female	13,295,555 (57.5%)	243,167 (41.7%)
Missing sex	2109 (<0.1%)	25 (<0.1%)
**Any hypertension diagnosis**		
No	10,214,051 (44.2%)	91,062 (15.6%)
Yes	12,916,468 (55.8%)	491,708 (84.4%)
**Diabetes mellitus**		
No	20,594,011 (89.0%)	495,878 (85.1%)
Yes	2,536,508 (11.0%)	86,892 (14.9%)
**Obesity**		
No	19,309,924 (83.5%)	470,007 (80.7%)
Yes	3,820,595 (16.5%)	112,763 (19.3%)
**Smoking/tobacco use**		
No	20,190,788 (87.3%)	495,314 (85.0%)
Yes	2,939,731 (12.7%)	87,456 (15.0%)
**Dyslipidemia**		
No	16,669,168 (72.1%)	267,693 (45.9%)
Yes	6,461,351 (27.9%)	315,077 (54.1%)
**Depression**		
No	20,419,347 (88.3%)	526,488 (90.3%)
Yes	2,711,172 (11.7%)	56,282 (9.7%)
**Anxiety disorders**		
No	20,461,117 (88.5%)	524,264 (90.0%)
Yes	2,669,402 (11.5%)	58,506 (10.0%)
**Bipolar disorder**		
No	22,575,475 (97.6%)	575,382 (98.7%)
Yes	555,044 (2.4%)	7388 (1.3%)

Values are unweighted discharge counts (column percentages) from all eligible discharges. Race and sex missingness are shown explicitly. Hypertension includes essential, secondary, resistant, and crisis hypertension and hypertensive heart or kidney disease. AF = atrial fibrillation; ACS = acute coronary syndrome; SD = standard deviation.

### 3.3 Associations between psychiatric disorders and atrial fibrillation

After adjustment, depression was associated with slightly higher odds of AF (adjusted OR, 1.041; 95% CI, 1.037–1.045; p < 0.001). Anxiety disorders (OR, 0.970; 95% CI, 0.966–0.974; p < 0.001) and bipolar disorder (OR, 0.848; 95% CI, 0.841–0.855; p < 0.001) were associated with lower odds of AF (Table 3).

**Table 3:** Survey-adjusted logistic regression for atrial fibrillation.

Characteristic	Adjusted OR	95% CI	p-value
Depression	1.041	1.037–1.045	<0.001
Anxiety disorders	0.970	0.966–0.974	<0.001
Bipolar disorder	0.848	0.841–0.855	<0.001
Any hypertension diagnosis	2.615	2.598–2.633	<0.001
Diabetes mellitus	0.848	0.844–0.851	<0.001
Obesity	1.005	1.000–1.009	0.064
Smoking/tobacco use	0.485	0.482–0.488	<0.001
Dyslipidemia	1.299	1.294–1.305	<0.001
Age 40–90 (18–39 reference)	19.358	19.010–19.712	<0.001
Female (male reference)	0.795	0.793–0.798	<0.001
Black (White reference)	0.488	0.484–0.492	<0.001
Hispanic	0.484	0.477–0.490	<0.001
Asian or Pacific Islander	0.654	0.643–0.665	<0.001
Native American	0.513	0.491–0.537	<0.001
Other race	0.615	0.600–0.630	<0.001
2018 (2017 reference)	1.042	1.030–1.055	<0.001
2019	1.064	1.052–1.077	<0.001
2020	1.066	1.054–1.079	<0.001

Survey-weighted complex-sample logistic regression using DISCWT, year-specific strata, and year-specific hospital clusters. Unweighted analytic N = 23,068,920. All listed variables were entered simultaneously; the reference category for binary variables is absence. Hypertension includes essential, secondary, resistant, and crisis hypertension and hypertensive heart or kidney disease. OR = odds ratio; CI = confidence interval.

Adults aged 40–90 years had markedly higher adjusted odds of AF than adults aged 18–39 years (OR, 19.358; 95% CI, 19.010–19.712). Hypertension was strongly associated with AF (OR, 2.615; 95% CI, 2.598–2.633). Dyslipidemia was also positively associated, whereas diabetes mellitus and smoking were inversely associated; obesity was not significant. Female sex and non-White race or ethnicity were associated with lower adjusted odds of AF than the reference groups (Table 3).

### 3.4 Age-stratified analysis of atrial fibrillation

Among adults aged 18–39 years, depression (OR, 1.149; 95% CI, 1.105–1.194) and anxiety disorders (OR, 1.095; 95% CI, 1.053–1.139) were associated with higher adjusted odds of AF. Bipolar disorder was not significantly associated with AF (OR, 1.009; 95% CI, 0.941–1.081; p = 0.805) (Table 5).

Among adults aged 18–39 years, hypertension had the strongest association with AF (OR, 4.012; 95% CI, 3.889–4.138). Obesity and dyslipidemia were also positively associated, whereas smoking was inversely associated and diabetes mellitus was not significant. Female sex was associated with substantially lower odds, Black race with higher odds, and Hispanic, Asian/Pacific Islander, and Other race with lower odds relative to White race (Table 5).

### 3.5 Association between psychiatric disorders and acute coronary syndrome

Depression (OR, 0.711; 95% CI, 0.704–0.718), anxiety disorders (OR, 0.862; 95% CI, 0.854–0.870), and bipolar disorder (OR, 0.504; 95% CI, 0.492–0.516) were each associated with lower adjusted odds of ACS (all p < 0.001) (Table 4).

**Table 4:** Survey-adjusted logistic regression for acute coronary syndrome.

Characteristic	Adjusted OR	95% CI	p-value
Depression	0.711	0.704–0.718	<0.001
Anxiety disorders	0.862	0.854–0.870	<0.001
Bipolar disorder	0.504	0.492–0.516	<0.001
Any hypertension diagnosis	2.103	2.080–2.126	<0.001
Diabetes mellitus	0.949	0.941–0.958	<0.001
Obesity	1.059	1.049–1.069	<0.001
Smoking/tobacco use	1.315	1.302–1.328	<0.001
Dyslipidemia	1.854	1.838–1.872	<0.001
Age 40–90 (18–39 reference)	6.579	6.429–6.732	<0.001
Female (male reference)	0.690	0.686–0.695	<0.001
Black (White reference)	0.824	0.810–0.838	<0.001
Hispanic	0.963	0.943–0.983	<0.001
Asian or Pacific Islander	1.158	1.120–1.197	<0.001
Native American	1.027	0.971–1.086	0.356
Other race	1.065	1.024–1.107	0.002
2018 (2017 reference)	0.827	0.802–0.852	<0.001
2019	0.807	0.782–0.832	<0.001
2020	0.809	0.784–0.835	<0.001

Survey-weighted complex-sample logistic regression using DISCWT, year-specific strata, and year-specific hospital clusters. Unweighted analytic N = 23,068,920. All listed variables were entered simultaneously; the reference category for binary variables is absence. Hypertension includes essential, secondary, resistant, and crisis hypertension and hypertensive heart or kidney disease. OR = odds ratio; CI = confidence interval.

**Table 5:** Survey-adjusted logistic regression for atrial fibrillation among discharges aged 18–39 Years.

Characteristic	Adjusted OR	95% CI	p-value
Depression	1.149	1.105–1.194	<0.001
Anxiety disorders	1.095	1.053–1.139	<0.001
Bipolar disorder	1.009	0.941–1.081	0.805
Any hypertension diagnosis	4.012	3.889–4.138	<0.001
Diabetes mellitus	0.994	0.942–1.048	0.819
Obesity	1.845	1.790–1.902	<0.001
Smoking/tobacco use	0.883	0.855–0.912	<0.001
Dyslipidemia	1.517	1.455–1.582	<0.001
Female (male reference)	0.240	0.233–0.248	<0.001
Black (White reference)	1.123	1.088–1.159	<0.001
Hispanic	0.744	0.714–0.776	<0.001
Asian or Pacific Islander	0.864	0.795–0.938	<0.001
Native American	0.916	0.803–1.046	0.195
Other race	0.827	0.769–0.889	<0.001
2018 (2017 reference)	1.038	0.992–1.086	0.103
2019	1.088	1.039–1.139	<0.001
2020	1.060	1.013–1.109	0.011

Survey-weighted domain analysis for discharges aged 18–39 years (unweighted domain N = 5,481,546; full-design analytic N = 23,068,920). The model simultaneously includes all listed covariates. Hypertension includes essential, secondary, resistant, and crisis hypertension and hypertensive heart or kidney disease. OR = odds ratio; CI = confidence interval.

Hypertension was strongly associated with ACS (OR, 2.103; 95% CI, 2.080–2.126). Obesity, smoking, and dyslipidemia were also associated with higher odds, whereas diabetes mellitus was inversely associated. Adults aged 40–90 years had higher odds than adults aged 18–39 years (OR, 6.579; 95% CI, 6.429–6.732), and female sex was associated with lower odds. Relative to White race, Black and Hispanic race or ethnicity had lower odds, Asian/Pacific Islander and Other race had higher odds, and Native American race was not significant (Table 4).

### 3.6 Age-stratified analysis of acute coronary syndrome

Among adults aged 18–39 years, depression (OR, 0.756; 95% CI, 0.705–0.810), anxiety disorders (OR, 0.892; 95% CI, 0.835–0.952), and bipolar disorder (OR, 0.626; 95% CI, 0.548–0.715) were associated with lower adjusted odds of ACS (Table 6).

**Table 6:** Survey-adjusted logistic regression for acute coronary syndrome among discharges aged 18–39 Years.

Characteristic	Adjusted OR	95% CI	p-value
Depression	0.756	0.705–0.810	<0.001
Anxiety disorders	0.892	0.835–0.952	<0.001
Bipolar disorder	0.626	0.548–0.715	<0.001
Any hypertension diagnosis	5.086	4.835–5.350	<0.001
Diabetes mellitus	1.032	0.962–1.106	0.383
Obesity	1.636	1.567–1.708	<0.001
Smoking/tobacco use	1.829	1.752–1.910	<0.001
Dyslipidemia	4.451	4.226–4.687	<0.001
Female (male reference)	0.362	0.345–0.379	<0.001
Black (White reference)	1.095	1.045–1.146	<0.001
Hispanic	0.891	0.841–0.944	<0.001
Asian or Pacific Islander	1.056	0.943–1.183	0.343
Native American	0.872	0.718–1.060	0.170
Other race	1.045	0.944–1.157	0.397
2018 (2017 reference)	0.761	0.710–0.815	<0.001
2019	0.792	0.741–0.848	<0.001
2020	0.800	0.746–0.858	<0.001

Survey-weighted domain analysis for discharges aged 18–39 years (unweighted domain N = 5,481,546; full-design analytic N = 23,068,920). The model simultaneously includes all listed covariates. Hypertension includes essential, secondary, resistant, and crisis hypertension and hypertensive heart or kidney disease. OR = odds ratio; CI = confidence interval.

Hypertension showed the strongest association with ACS among adults aged 18–39 years (OR, 5.086; 95% CI, 4.835–5.350), followed by dyslipidemia, smoking, and obesity. Diabetes mellitus was not significant. Female sex was inversely associated with ACS. Black race was positively associated and Hispanic race inversely associated relative to White race; Asian/Pacific Islander, Native American, and Other race were not significant (Table 6).

### 3.7 Interactions between age group and psychiatric disorders or hypertension

Interaction models assessed whether age group modified the associations of depression, anxiety disorders, bipolar disorder, and hypertension with AF and ACS.

For AF, the age-group interactions were significant for depression (interaction OR, 0.916; 95% CI, 0.882–0.953), anxiety disorders (0.930; 0.894–0.967), bipolar disorder (0.810; 0.756–0.868), and hypertension (0.312; 0.304–0.321), all p < 0.001 (Table 7). For ACS, the interactions were significant for depression (0.894; 0.834–0.958; p = 0.001), bipolar disorder (0.745; 0.652–0.852; p < 0.001), and hypertension (0.200; 0.192–0.208; p < 0.001), but not anxiety disorders (0.951; 0.890–1.016; p = 0.134) (Table 8). Hypertension remained positively associated with both outcomes in each age group, although the association was stronger among adults aged 18–39 years.

**Table 7:** Age-interaction model for atrial fibrillation.

Characteristic	Adjusted OR	95% CI	p-value
Depression (age 18–39 effect)	1.134	1.092–1.179	<0.001
Anxiety disorders (age 18–39 effect)	1.042	1.003–1.084	0.037
Bipolar disorder (age 18–39 effect)	1.044	0.974–1.118	0.222
Any hypertension diagnosis (age 18–39 effect)	8.216	7.997–8.442	<0.001
Age 40–90 main effect	29.356	28.712–30.015	<0.001
Age × depression	0.916	0.882–0.953	<0.001
Age × anxiety disorders	0.930	0.894–0.967	<0.001
Age × bipolar disorder	0.810	0.756–0.868	<0.001
Age × hypertension	0.312	0.304–0.321	<0.001

Survey-weighted interaction model using the full analytic sample (unweighted N = 23,068,920). The main psychiatric and hypertension effects represent ages 18–39. The age 40–90 main effect represents the age-group association when the interacting exposures are absent. Interaction ORs are ratios of the corresponding OR in ages 40–90 to that in ages 18–39. The model also adjusts for diabetes, obesity, smoking, dyslipidemia, sex, race, and year. OR = odds ratio; CI = confidence interval.

**Table 8:** Age-interaction model for acute coronary syndrome.

Characteristic	Adjusted OR	95% CI	p-value
Depression (age 18–39 effect)	0.793	0.741–0.850	<0.001
Anxiety disorders (age 18–39 effect)	0.904	0.847–0.966	0.003
Bipolar disorder (age 18–39 effect)	0.669	0.586–0.763	<0.001
Any hypertension diagnosis (age 18–39 effect)	9.861	9.484–10.252	<0.001
Age 40–90 main effect	12.820	12.414–13.240	<0.001
Age × depression	0.894	0.834–0.958	0.001
Age × anxiety disorders	0.951	0.890–1.016	0.134
Age × bipolar disorder	0.745	0.652–0.852	<0.001
Age × hypertension	0.200	0.192–0.208	<0.001

Survey-weighted interaction model using the full analytic sample (unweighted N = 23,068,920). The main psychiatric and hypertension effects represent ages 18–39. The age 40–90 main effect represents the age-group association when the interacting exposures are absent. Interaction ORs are ratios of the corresponding OR in ages 40–90 to that in ages 18–39. The model also adjusts for diabetes, obesity, smoking, dyslipidemia, sex, race, and year. OR = odds ratio; CI = confidence interval.

### 3.8 Summary of findings

This study yielded three principal findings. First, depression was associated with slightly higher odds of AF, whereas anxiety disorders and bipolar disorder were associated with lower odds; all three psychiatric exposures were associated with lower odds of ACS. Second, among adults aged 18–39 years, depression and anxiety disorders were positively associated with AF, bipolar disorder was not significant, and all three psychiatric exposures were inversely associated with ACS. Third, hypertension was strongly associated with both outcomes, and its association was greater among adults aged 18–39 years. Age interactions were also present for each psychiatric exposure in the AF model and for depression and bipolar disorder, but not anxiety, in the ACS model.

## 4 Discussion

### 4.1 Principal findings

In the fully adjusted analysis, depression was associated with a small increase in the odds of recorded atrial fibrillation (AF), whereas anxiety and bipolar disorder were associated with lower odds. All three psychiatric conditions were inversely associated with recorded acute coronary syndrome (ACS). In contrast, hypertension showed a strong positive association with both cardiovascular outcomes. Several associations varied by age. Among the age-interaction terms examined in the ACS model, only the interaction with anxiety was not statistically significant.

Age-related differences were most pronounced for AF. Among adults aged 18–39 years, depression and anxiety were associated with higher odds of recorded AF, whereas bipolar disorder was not significantly associated. In the younger ACS subgroup, depression, anxiety, and bipolar disorder were each associated with lower odds of the recorded outcome. Hypertension was positively associated with both AF and ACS, with stronger associations among adults aged 18–39 years. The interaction analyses supported age-related variation in most psychiatric associations; however, there was no evidence that the association between anxiety and ACS differed significantly between age groups.

These findings should be interpreted in the context of the study's large sample size. For example, the associations of depression and anxiety with AF in the overall cohort were small in magnitude (odds ratios 1.041 and 0.970, respectively), despite narrow confidence intervals and statistical significance. The age-restricted and interaction analyses indicate heterogeneity across age groups; however, these estimates remain subject to selection effects, coding practices, and residual confounding inherent in cross-sectional inpatient data.

### 4.2 Psychiatric disorders and atrial fibrillation

A growing body of evidence demonstrates an association between psychiatric disorders and AF. Large prospective cohort studies and systematic reviews consistently report that depression and anxiety disorders are linked to an increased incidence of AF, even after adjustment for conventional cardiovascular risk factors [6,13]. Proposed mechanisms include chronic activation of the sympathetic nervous system, dysregulation of the hypothalamic–pituitary–adrenal axis, endothelial dysfunction, oxidative stress, systemic inflammation, and atrial structural remodeling, each of which may increase susceptibility to atrial arrhythmias [3,9,11,12].

The full-cohort findings differed from most longitudinal studies: depression was associated with slightly higher odds of AF, whereas anxiety disorders and bipolar disorder were associated with lower odds. These hospitalization-level associations should not be interpreted as effects on incident AF or as evidence of protection.

Among adults aged 18–39 years, depression and anxiety disorders were associated with higher odds of AF, while bipolar disorder was not significant. Significant age interactions for all three psychiatric exposures further support heterogeneity between younger and older hospitalized adults, although the cross-sectional design prevents causal interpretation.

### 4.3 Psychiatric disorders and acute coronary syndrome

Depression is recognized as an independent predictor of adverse cardiovascular outcomes following myocardial infarction and is linked to an increased risk of coronary artery disease, recurrent ischemic events, and cardiovascular mortality [3,5]. Anxiety disorders are also associated with coronary artery disease through mechanisms such as sympathetic activation, endothelial dysfunction, platelet activation, and chronic inflammation, although the strength of these associations varies across studies [6,7]. Similarly, bipolar disorder is consistently associated with premature cardiovascular disease and reduced life expectancy, reflecting both traditional cardiovascular risk factors and the metabolic effects of psychotropic medications [8].

Depression, anxiety disorders, and bipolar disorder were each associated with lower adjusted odds of ACS in the full cohort and among adults aged 18–39 years. These inverse hospitalization-level associations should not be interpreted as protective effects and may reflect differences in admission patterns, diagnostic documentation, healthcare utilization, and residual confounding.

Age-stratified analyses demonstrated that conventional cardiovascular risk factors remained important predictors of ACS among younger adults. Hypertension, dyslipidemia, smoking, and obesity were positively associated with ACS in this subgroup, whereas diabetes mellitus was not independently associated. These findings align with previous studies indicating that premature coronary artery disease is primarily driven by modifiable cardiovascular risk factors and support current guideline recommendations emphasizing early and aggressive risk factor modification [14,15].

The diabetes-related findings warrant separate consideration. In the unadjusted, age-stratified analyses, the prevalence of recorded ACS was higher among hospitalizations involving diabetes in both age groups: 0.8% versus 0.2% among adults aged 18–39 years and 3.4% versus 3.1% among adults aged 40–90 years. After simultaneous adjustment for hypertension, dyslipidemia, obesity, smoking, demographic characteristics, and psychiatric comorbidity, diabetes was not independently associated with ACS among adults aged 18–39 years and showed a small inverse association in the overall cohort. This change in direction should not be interpreted as evidence that diabetes is protective. Instead, it represents the association remaining after accounting for correlated cardiometabolic factors and may also reflect adjustment for variables along shared disease pathways, selection within the hospitalized population, and differences in diagnostic coding. Because diabetes was included as an adjustment covariate rather than as a primary exposure, its coefficient should be interpreted cautiously and should not be considered an estimate of the total cardiovascular effect of diabetes.

### 4.4 Interpretation of the inverse associations

The inverse associations observed between psychiatric disorders and both AF and ACS require cautious interpretation. These findings do not indicate that depression, anxiety disorders, or bipolar disorder confer protection against cardiovascular disease. Instead, they likely reflect methodological and clinical characteristics inherent to hospitalization-based administrative data, differences in study design, and residual confounding that distinguish the present analysis from prospective cohort studies of incident cardiovascular disease.

The comparison group included hospitalized patients who did not have a recorded diagnosis of depression, anxiety disorder, or bipolar disorder, so they should not be seen as a healthy control group. These patients were admitted for various medical and surgical reasons. They may have differed from those with documented psychiatric disorders in terms of hospitalization reasons, comorbidities, healthcare utilization, and extent of diagnostic evaluation. Some psychiatric disorders might not have been recorded. These factors could have affected whether AF or ACS was identified and documented, which may help partially explain the associations we observed.

A key consideration is the structure of the NIS. Unlike longitudinal cohort studies that track individuals over time, the NIS records diagnoses from a single hospitalization event. As a result, this analysis estimates cross-sectional associations between coexisting psychiatric and cardiovascular diagnoses rather than assessing the future risk of developing AF or ACS. Patients admitted primarily for psychiatric illness differ markedly from those hospitalized for acute cardiovascular conditions in terms of age, comorbidity burden, healthcare utilization, and reasons for admission. These differences may influence diagnostic evaluation and coding practices, thereby affecting the observed associations [16,17].

Residual confounding also warrants consideration. Although the analyses adjusted for several established cardiovascular risk factors, including hypertension, diabetes mellitus, obesity, smoking, dyslipidemia, age, sex, and race or ethnicity, important clinical variables were not available in the NIS. Data on medication use, duration and severity of psychiatric illness, outpatient treatment, alcohol consumption, physical activity, socioeconomic status, family history of cardiovascular disease, and laboratory findings could not be included. These unmeasured variables may influence both psychiatric diagnoses and cardiovascular outcomes and thus remain potential sources of residual confounding [18].

Overadjustment may also contribute to the observed associations. Several cardiovascular risk factors included in the multivariable models may be downstream consequences of psychiatric illness rather than independent confounders. Depression and anxiety are closely linked to smoking, obesity, diabetes mellitus, hypertension, physical inactivity, and poor adherence to preventive therapies, all of which increase cardiovascular disease risk [3,9]. Adjusting for these variables estimates the association between psychiatric disorders and cardiovascular outcomes independent of these intermediary pathways. This adjustment may have made the associations appear weaker, leading to lower adjusted odds ratios compared to studies that used other adjustment methods. These estimates show the associations after considering the included cardiovascular risk factors and should not be interpreted as representing the overall cardiovascular impact of psychiatric disorders.

Variations in diagnostic coding may contribute to the observed findings. Administrative databases depend on ICD-10-CM coding for identification of both exposure and outcomes. During hospitalizations for acute cardiovascular conditions, clinical documentation typically prioritizes the primary cardiac diagnosis, resulting in incomplete documentation of chronic psychiatric disorders. In contrast, patients admitted primarily for psychiatric illness often receive less intensive cardiovascular evaluation unless clinically indicated, increasing the likelihood of underrecognition of asymptomatic or less severe cardiovascular disease. This differential coding is recognized as an inherent limitation of administrative databases and may affect observed associations [16].

Age-domain and interaction analyses demonstrated heterogeneity for both outcomes. Among adults aged 18–39 years, depression and anxiety disorders were positively associated with AF, whereas all three psychiatric exposures were inversely associated with ACS. For AF, the interactions with age were significant for depression, anxiety disorders, bipolar disorder, and hypertension. For ACS, the interactions were significant for depression, bipolar disorder, and hypertension, but not anxiety disorders. These findings indicate that estimates from the combined adult population do not fully represent age-specific associations.

### 4.5 Clinical implications

The present findings have several important clinical implications. First, they indicate that associations identified within hospitalization-based administrative databases require interpretation distinct from estimates derived from prospective cohort studies. Cross-sectional inpatient analyses offer valuable insight into disease burden, healthcare utilization patterns, and the coexistence of medical conditions. However, these analyses are not designed to establish temporal relationships or causality. Therefore, these findings should not prompt changes to current recommendations regarding cardiovascular risk assessment in patients with psychiatric disorders.

The age-domain and interaction analyses underscore the importance of considering age when interpreting associations between psychiatric and cardiovascular diagnoses. The positive associations of depression and anxiety disorders with AF among adults aged 18–39 years contrast with the full-cohort pattern and support further investigation of age-specific mechanisms.

Finally, the strong associations of hypertension, dyslipidemia, obesity, and smoking with AF or ACS among younger adults reinforce the importance of early and aggressive cardiovascular risk-factor modification. Diabetes mellitus was not independently associated with either outcome in this subgroup. These observations align with current cardiovascular prevention guidelines and highlight the need for comprehensive cardiovascular risk assessment among patients with psychiatric disorders [14,15].

Cardiovascular care for patients with psychiatric disorders should continue to emphasize established preventive measures, including smoking cessation, regular physical activity, healthy dietary patterns, weight management, adequate sleep, and appropriate control of blood pressure, diabetes mellitus, and dyslipidemia [14,15].

### 4.6 Strengths

This study demonstrates several notable strengths. First, it employs the HCUP NIS, the largest publicly available all-payer inpatient database in the United States, which offers a nationally representative sample of hospitalized adults and robust statistical power. Second, in contrast to many previous investigations, this analysis simultaneously evaluates depression, anxiety disorders, and bipolar disorder within the same multivariable models, enabling estimation of the independent association of each psychiatric disorder while accounting for psychiatric comorbidity. Third, adjustment for multiple established cardiovascular risk factors minimizes confounding from traditional cardiovascular disease determinants. Finally, the inclusion of prespecified age-stratified and interaction analyses yields additional insight into age-related heterogeneity that would not have been evident from the overall analyses alone.

### 4.7 Limitations

Several limitations must be acknowledged when interpreting these findings. First, the cross-sectional design prevents determination of temporal relationships and does not allow causal inference between psychiatric disorders and cardiovascular outcomes. Second, the NIS is an administrative database that depends on ICD-10-CM coding, making it vulnerable to coding errors and misclassification. Third, because the unit of analysis is the hospitalization rather than the individual patient, repeat admissions by the same patient cannot be distinguished. Fourth, several clinically relevant variables, such as medication use, laboratory values, disease severity, outpatient treatment, duration of psychiatric illness, lifestyle factors, and family history, were unavailable, which may result in residual confounding. Finally, although the NIS provides nationally representative inpatient data, these findings may not be generalizable to outpatient or community-based populations.

## 5 Conclusion

This nationally representative cross-sectional analysis of hospitalizations among adults aged 18–90 years found outcome- and age-dependent associations between psychiatric disorders and cardiovascular diagnoses. In the full models, depression was associated with slightly higher odds of AF, whereas anxiety disorders and bipolar disorder were associated with lower odds; depression, anxiety disorders, and bipolar disorder were each associated with lower odds of ACS. Hypertension was strongly associated with both cardiovascular outcomes. Among adults aged 18–39 years, depression and anxiety disorders were positively associated with AF, bipolar disorder was not significant, and all three psychiatric exposures were inversely associated with ACS. Age interactions were significant for each psychiatric exposure and hypertension in the AF model; in the ACS model, the anxiety interaction was not significant.

These findings show that the relationship between psychiatric disorders and cardiovascular diagnoses is complex and may differ by age and clinical setting. Because this study used cross-sectional hospitalization-level data, the results reflect psychiatric and cardiovascular diagnoses recorded during the same hospital stay rather than new cardiovascular disease developing over time. Therefore, the findings cannot determine which condition occurred first, establish causality, or suggest that psychiatric disorders protect against cardiovascular disease. The comparison group included hospitalized patients without a recorded psychiatric diagnosis and should not be viewed as a healthy control group. Variations in reasons for admission, diagnostic evaluation, coding practices, and healthcare utilization may have influenced the observed associations. The results should be interpreted with consideration of adjustments for potential intermediate pathways and residual confounding. Longitudinal patient-level studies incorporating more detailed clinical data are needed to clarify the timing and mechanisms underlying the relationship between psychiatric disorders and cardiovascular disease.

## CRediT authorship contribution statement

**Abdelrazig Suliman:** Writing – review & editing, Writing – original draft, Software, Investigation, Formal analysis. **Fayz Quadri:** Writing – review & editing, Writing – original draft. **Gloria Elizondo:** Writing – review & editing, Writing – original draft. **Harshil Patel:** Writing – original draft. **Abdullah Rabah:** Writing – review & editing, Writing – original draft, Supervision. **Chi Ayrsh Ford:** Writing – review & editing, Writing – original draft, Methodology.

## Ethics approval

Not required (use of publicly available de-identified HCUP NIS data)

## Declaration of generative AI and AI-assisted technologies in the manuscript preparation process

During the revision of this manuscript, the authors used OpenAI ChatGPT and Codex to assist with language editing, content organization, consistency checks, and the presentation of author-generated statistical results. The statistical analyses were conducted by the authors using SPSS. The authors independently reviewed and verified all numerical results, references, interpretations, tables, and conclusions, revised the AI-assisted material as needed, and take full responsibility for the content of the manuscript.

## Funding

None

## Declaration of competing interests

The authors declare no conflicts of interest.
