# Supplementary material for: Independent associations of psychiatric disorders with atrial fibrillation and acute coronary syndrome: An analysis of the National Inpatient Sample
Source: Int J Cardiol Cardiovasc Risk Prev. 2026 Aug 24;31:200704. doi: 10.1016/j.ijcrp.2026.200704 (PMC13577834; doi:10.1016/j.ijcrp.2026.200704)
Supplement: Supplementary file 1 [file mmc1.docx]

**Supplementary Table 1**

**ICD-10-CM Codes Used to Define Study Variables**

| **Variable** | **ICD-10-CM code(s)** |
| --- | --- |
| Depression | F32, F33, F34.1 |
| Anxiety disorders | F40, F41 |
| Bipolar disorder | F31 |
| Atrial fibrillation | I48.0, I48.1, I48.2, I48.9 |
| Acute coronary syndrome | I20.0, I21, I22 |
| Hypertension | I10–I13, I15–I16, I1A.0 |
| Diabetes mellitus | E08, E09, E10, E11, E13 |
| Obesity | E66 |
| Smoking or tobacco use | F17, Z72.0, Z87.891 |
| Dyslipidemia | E78 |

Abbreviation: ICD-10-CM = International Classification of Diseases, Tenth Revision, Clinical Modification.
